# Supplementary material for: Inhibition of Human Cytomegalovirus Entry into Host Cells through A Pleiotropic Small Molecule
Source: Int J Mol Sci. 2020 Feb 29;21(5):1676. doi: 10.3390/ijms21051676 (PMC7084493; doi:10.3390/ijms21051676)
Supplement: Supplementary file 1 [file ijms-21-01676-s001.pdf]

Inhibition of Human Cytomegalovirus Entry into Host Cells Through a Pleiotropic Small Molecule

James Elste[1†], Dominik Kaltenbach[2†], Vraj R Patel[1], Max T Nguyen[1], Harsh Sharthiya[3], Ritesh Tandon[4], Satish K Mehta[5], Michael V Volin[1], Michele Fornaro[3], Vaibhav Tiwari[1]*, Umesh R Desai[6,7]*

[1]*Department of Microbiology & Immunology, College of Graduate Studies and Chicago College of Osteopathic Medicine, Midwestern University, Downers Grove, IL 60515, USA*

[2]*Department of Biomedical Sciences, College of Graduate Studies, Midwestern University, Downers Grove, IL 60515, USA*

[3]*Department of Anatomy, College of Graduate Studies and Chicago College of Osteopathic Medicine, Midwestern University, Downers Grove, IL 60515, USA*

[4]*Department of Microbiology and Immunology, University of Mississippi Medical Center, 2500 North State Street, Jackson, MS 39216, USA.*

[5] *KBR Wyle Laboratories, Houston, TX* 77058, *USA.*

[6]*Department of Medicinal Chemistry, School of Pharmacy, Virginia Commonwealth University, Richmond, VA 23298 USA*

and

[7]*Institute for Structural Biology, Drug Discovery and Development, Virginia Commonwealth University, Richmond, VA 23219 USA*

---

* Corresponding authors. (Tiwari) vtiwar@midwestern.edu; (Desai) urdesai@vcu.edu

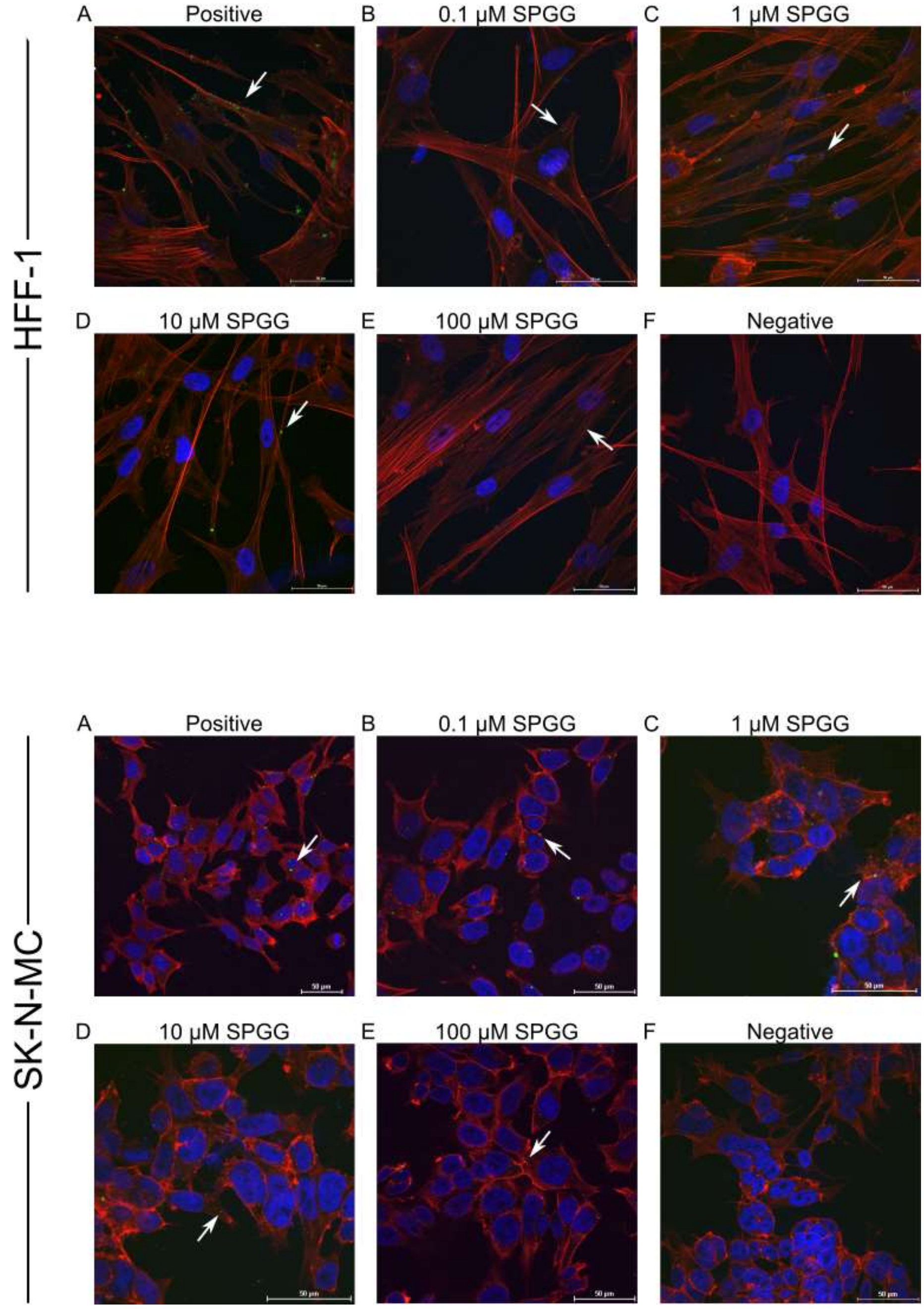

Figure S1. Localization of HCMV entry into HFF-1 and SK-N-MC cells treated with SPGG in High-resolution confocal microscopy. Full panels. This figure is an extended dose-dependent view of Fig. 2 showing double immunostained HFF-1 (top panel) and SK-N-MC (lower panel) cells with HCMV-GFP (green), phalloidin (red actin stain), and DAPI (blue nuclear stain) markers. Both cell lines were challenged with the HCMV strain BAD32GFP pre-incubated with different concentrations of SPGG for 1 hour. Numerous GFP-positive puncta were detectable in both cell lines untreated with SPGG (A, Positive control). In both models, a decreased number of GFP-positive puncta was observed in correlation with incremental concentrations of SPGG (B-E). No GFP labeling were observed in cells samples mock infected with HCMV (F, negative control). Scale bars (A-F) = 50μm.
